# Cascaded Deep Learning-Based Model for Classification and Segmentation of Plaques from Carotid Ultrasound Images

**DOI:** 10.3390/bioengineering13020190

**Published:** 2026-02-06

**Authors:** Bo-Wen Ren, Ran Zhou, Xinyao Cheng, Mingyue Ding, Bernard Chiu

**Affiliations:** 1 Department of Electrical Engineering, City University of Hong Kong, Hong Kong Special Administrative Region, China; bowenren5-c@my.cityu.edu.hk; 2 School of Computer Science and Artificial Intelligence, Hubei University of Technology, Wuhan 430068, China; ranzhou@hbut.edu.cn; 3 Department of Cardiology, Zhongnan Hospital, Wuhan University, Wuhan 430071, China; xycheng@whu.edu.cn; 4 Department of Biomedical Engineering, College of Life Science and Technology, Huazhong University of Science and Technology, Wuhan 430074, China; myding@hust.edu.cn; 5 Advanced Bio-Medical Imaging Facility, Huazhong University of Science and Technology, Wuhan 430074, China; 6 Department of Computer Science & Physics, Wilfrid Laurier University, Waterloo, ON N2L 3C5, Canada

**Keywords:** carotid plaque, ultrasound, classification, segmentation

## Abstract

Carotid plaque classification based on ultrasound echogenicity and quantification of plaque burden are crucial in stroke risk assessment. In this work, we propose a framework that leverages the synergy between classification and segmentation by sharing plaque location information to enhance the performance of both tasks. Our cascaded framework integrates a ResNet-based classifier (Masked-ResNet-DS) with MedSAM, a medically adapted version of the Segment Anything Model for joint classification and segmentation of carotid plaques from 2D ultrasound images. Ground truth boundaries are used to guide region-specific feature pooling in the classifier, helping it focus on plaques during training. Since ground truth boundaries are unavailable at inference, we introduce a two-iteration strategy: the first generates a class activation map (CAM), which is then used for focused pooling in the second iteration to predict plaque type. The CAM is also used as a prompt to guide MedSAM for segmentation. To ensure accurate localization, the CAM is supervised during training using a Dice loss against the segmentation ground truth. Masked-ResNet-DS achieves a mean F1-score of 96.7% in plaque classification, at least 3.2% higher than competing methods. Ablation studies confirm that ground truth-based pooling and CAM supervision both improve classification. CAM-guided MedSAM achieves a Dice similarity coefficient (DSC) of 86.6%, outperforming U-Net and nnU-Net by 5.9% and 3.6%, respectively. In addition, CAM prompts improve MedSAM’s DSC by 2.2%. By sharing plaque location between classification and segmentation, the proposed method improves both tasks and provides a more accurate tool for stroke risk stratification.

## 1. Introduction

Stroke was the second leading cause of death globally in 2021, following ischemic heart disease [1]. The prevalence of cardiovascular diseases, including stroke, nearly doubled from 271 million cases in 1990 to 523 million in 2019 [2]. Strokes are predominantly ischemic, and carotid is a major source of emboli leading to ischemic strokes. Therefore, monitoring carotid atherosclerosis plays an important role in risk stratification, stroke prevention and evaluation of novel therapies. Among key factors affecting stroke risk are plaque composition [3] and progression/regression of plaque burden [4]. Carotid ultrasound is useful in characterizing plaque composition based on its echogenic properties [5] and in quantifying the temporal changes in plaque burden by total plaque area (TPA) measurement [4]. Investigations of plaque echogenic properties involve classifying plaques into specific types based on their echogenicity and TPA measurement, requiring delineation of plaques as detailed below.

Plaque classification based on the ultrasound echogenicity is important in evaluating carotid artery plaque vulnerability, which is critical for stroke risk assessment [6]. The ultrasound echogenicity of the carotid plaques is reported to be inversely related to the soft tissue contents of the plaques [7]. Echolucent plaques, characterized by low ultrasound reflectivity, are more vulnerable due to their composition of large lipid cores, thin fibrous caps, intraplaque hemorrhage, and minimal calcification, increasing the risk of rupture and subsequent cerebrovascular events [8,9]. On the other hand, echogenic plaques, primarily composed of calcifications and fibrotic tissue, are more stable [10,11]. Classifying plaques into echogenic (EG), intermediate (IM), and echolucent (EL) categories based on B-mode ultrasound appearance provides critical insights into their composition, which enables non-invasive risk of plaque rupture stratification for cerebrovascular events [12,13,14]. However, subjective plaque echogenicity classification from ultrasound images was reported to be less reproducible due to observer dependency [15]. Therefore, echogenicity classification remains challenging, and advanced automatic algorithms are required.

Plaque segmentation is required for quantifying plaque burden from carotid ultrasound images and allows the measurement of total plaque area (TPA), which is a powerful predictor of risk [4,16]. Accurate TPA measurement requires precise localization and delineation of plaques in ultrasound images, which, if performed manually, are time-consuming, labor-intensive and operator-dependent. This underscores the importance of developing automated systems for carotid plaque segmentation to enhance efficiency, reduce variability, and improve stroke risk stratification in clinical practice.

Convolutional neural networks (CNNs) have been developed for carotid plaque classification and segmentation. For plaque classification, Lekadir et al. proposed a CNN for carotid plaque classification to automatically characterize plaque composition [17]. Ma et al. introduced a multilevel strip pooling module to modify the conventional VGG network for classifying plaque echogenicity [18]. Saba et al. proposed a deep learning algorithm to classify carotid plaques into asymptomatic and asymptomatic categories [19]. For plaque segmentation, Zhou et al. trained a modified U-Net model to segment carotid plaques for TPA measurement [20]. They also developed a UNet++ model to increase the generalizability of the network, such that the model can be trained on a small dataset and applied to an independent dataset without retraining [21]. While these studies demonstrate progress in individual tasks, they did not leverage the synergy of classification and segmentation, thereby limiting further performance improvement in individual tasks.

There have been approaches developed to integrate classification and segmentation of skin lesions from dermoscopic images. Yu et al. [22] developed a two-stage framework, with a CNN segmenting the skin lesion, followed by a second CNN for skin lesion type classification. However, there was no interaction between the segmentation and classification CNNs, except that the segmented lesion was used as the input to the classification network. Xie et al. [23] developed a network known as the mutual bootstrapping deep convolutional neural network (MB-DCNN) for skin lesion classification and segmentation, in which a coarse segmentation network generates an initial lesion mask to be concatenated with the input image to enhance the classification accuracy of a mask-guided classification network. The classification network then predicts the lesion type and outputs a class activation map (CAM) highlighting the region of interest (ROI). The CAM is then fed to the enhanced segmentation network as an input feature map to generate a final segmentation contour. This approach, however, requires two segmentation networks, increasing model complexity and computational demand. Additionally, CAM supervised only by classification loss may only cover the most discriminative regions, instead of the entire object of interest [24]. Using this CAM for guidance may lower the performance of the final segmentation network. He et al. [25] proposed a joint framework with a shared encoder followed by three decoders dedicated to segmentation, classification and edge detection. Introducing the edge decoder solely for enhancing the performance of the segmentation decoder increases the complexity of overall architecture. Moreover, if the edge map generated by the edge decoder is inaccurate, the segmentation decoder’s reliance on it may introduce an additional source of error.

In this work, we propose a cascaded framework for carotid plaque classification and segmentation from 2D ultrasound images. The framework consists of a ResNet-based classifier and the MedSAM—a medically adapted version of the Segment Anything Model (SAM)—for segmentation, designed to enable synergistic information flow between the two tasks. The classification network focuses on identifying plaque echogenicity, while the class activation map (CAM) it generates serves as a mask prompt for MedSAM. To ensure effective guidance, the CAM is encouraged to encompass the full plaque region.

As there has been concern about the reliability of CAM to be used as a localizer [24], we introduce a Dice loss between the CAM and the segmentation ground truth during classification training to improve localization accuracy and promote whole coverage of the plaque. This not only improves the CAM’s utility as a segmentation prompt but also helps the classifier attend more precisely to plaque regions. The integration of classification-driven prompts into MedSAM leverages its promptable capabilities, resulting in a simple and efficient dual-task pipeline.

Although MedSAM has demonstrated strong general segmentation capabilities, its performance when guided by CAM-based prompts—particularly in medical imaging—remains underexplored. This work addresses this gap by evaluating how CAM-guided prompting affects MedSAM’s performance on plaque segmentation, thereby advancing understanding of prompt-based segmentation in clinical applications.

## 2. Materials and Methods

### 2.1. Image Acquisition and Preprocessing

The dataset analyzed comprises a total of 1463 ultrasound images of carotid plaques acquired from 925 patients in a study at Zhongnan Hospital of Wuhan University, as described by Ma et al. [18]. The study was approved by the Institutional Review Board of the Medical School, Wuhan University, and written informed consent was obtained from all patients [18]. The images were acquired with an Acuson SC2000 (Siemens, Erlangen, Germany) US system equipment with a 5–12 MHz linear array probe (9L4). All subjects were examined in the supine position with the head tilted slightly backward. The ultrasound probe was placed perpendicular to the carotid vessel wall and moved downward from the carotid bifurcation. A longitudinal image (i.e., a long-axis view) was captured when a visible plaque was identified. Specific acquisition parameters, such as imaging depth and gain settings, varied across patients. Gain settings were adjusted for each patient to optimize image clarity. Plaque echogenicity was manually classified by an expert clinician according to the criteria specified by the European carotid plaque study group [7]. Each plaque was classified twice by the same observer in two sessions spaced three months apart to confirm high intraobserver agreement (κ=0.747) [18].

### 2.2. Network Architecture

**Figure 1:**
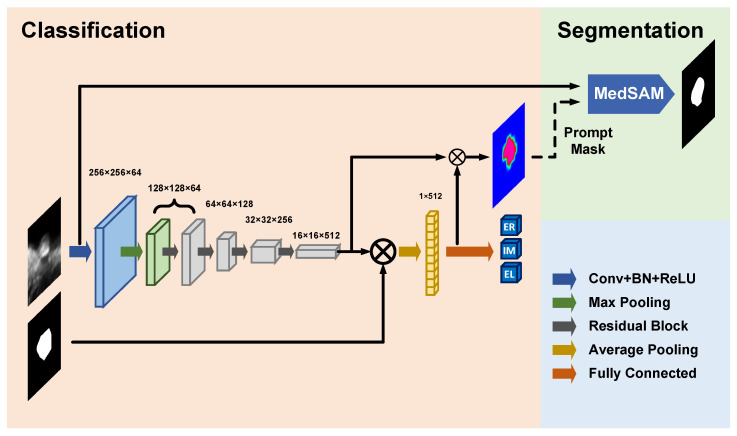
Schematic diagram of the cascaded carotid plaque classification and segmentation network. The classification and segmentation networks are enclosed by the peach and green rectangular boxes, respectively. Conv: Convolution, BN: Batch normalization, ReLU: Rectified linear unit.

Our framework combines a classification network for categorizing plaque types and a segmentation network for delineating plaque boundaries, with attention maps linking the two tasks, as depicted in Figure 1. The classification network categorizes plaques into three echogenicity classes—echogenic (EG), intermediate (IM), and echolucent (EL)—and generates class activation maps (CAMs) [26] highlighting plaque regions critical for classification. The CAM serves as a prompt for the segmentation network.

The classification network was built on a ResNet backbone, categorizing cropped 2D carotid ultrasound images of size 512×512 into the three classes. To allow better focus on plaque regions during training, the last feature map was masked with the ground truth segmentation before global average pooling. As the network was blinded to the ground truth of test images, the inference stage requires an extra iteration of the network to establish the focus before predicting the echogenicity class. Specifically, in the first iteration, the image was processed by the network without any masking in the pooling operation. In this iteration, the CAM was generated by weighting the last feature map using the fully connected (FC) layer’s weights corresponding to the predicted class. In the second iteration, CAM was applied to mask the last feature map before the global average pooling operation. Specifically, this was achieved by pixel-wise multiplication of the CAM and the last feature map.

The segmentation network employs the MedSAM [27] for precise segmentation, a vision transformer-based architecture designed for promptable segmentation. The SAM takes prompt points, boxes, or masks as optional input. In this work, the SAM takes the original images, segmentation ground truth (for loss computation), and the CAM as the input for both training and testing phases, with no bounding box and point inputs used. Input images were resized to the standard MedSAM resolution of 1024×1024 pixels. Following the official MedSAM fine-tuning protocol, the pre-trained vision transformer (ViT)-based image encoder was frozen, while the prompt encoder and lightweight mask decoder were trained based on the sum of binary cross entropy loss and Dice loss between algorithm and ground truth segmentation. The CAMs from the classification stage were used as mask prompts in both the training and testing stages of MedSAM. CAMs of the training images were used instead of the ground truth segmentations to ensure consistency of the prompts across the training and the testing stages, as ground truth segmentations are not available for the test images.

### 2.3. Loss Function

In the classification task, two loss functions are assigned in unweighted combination to balance classification accuracy and CAM quality:

(1) $$\begin{array}{cc} L_{\mathrm{cls}}= & -\sum_{i=1}^{3}y_{i}\log p_{i} \end{array}$$

(2) $$\begin{array}{cc} L_{\mathrm{Dice}}= & 1-\frac{2\sum_{j=1}^{M}f_{j}g_{j}}{\sum_{j=1}^{M}\left(f_{j}+g_{j}\right)+\epsilon } \end{array}$$

where $y_{i}\in \left\{0,1\right\}$ is the one-hot encoded true label ($y_{i}=1$ if the sample belongs to class *i*, and 0 otherwise), $p_{i}\in \left[0,1\right]$ is the predicted probability for class *i* via the softmax function, $f_{j}$ and $g_{j}$ are the *j*-th pixel values of the predicted CAM and ground truth segmentation masks, respectively, *M* is the image pixels number, and $\epsilon =1\times 10^{-5}$ prevents division by zero. The cross-entropy loss $L_{\mathrm{cls}}$ minimizes classification errors, while the Dice loss $L_{\mathrm{Dice}}$ aligns the predicted CAM with the segmentation ground truth, enhancing its utility for segmentation. We will discuss the effects of the Dice loss in the following sections. The MedSAM was trained using the unweighted combination of the Dice loss and the binary cross-entropy loss.

### 2.4. Evaluation and Statistical Analyses

In total, 1463 2DUS images of carotid plaques were acquired from 925 patients, from which 70% (1025 images), 10% (144 images), and 20% (294 images) were involved in training, validation, and testing, respectively. The data partitioning scheme is tabulated in Table 1, with the number of images used in each partition the same as in Ma et al. [18]. Multiple images acquired from the same subject were partitioned into the same set. In training, the validation set was used to optimize each model. For classification, the model achieving the highest classification accuracy on the validation set was considered optimal. For segmentation, the MedSAM was trained with original images, segmentation ground truths and CAM as the mask prompt. The model with the highest Dice Similarity Coefficient (DSC) on the validation set was selected as the optimal model.

In classification, the stochastic optimization algorithm Adam optimizer was adopted to minimize the loss function. The classification models were trained with the following parameters: the number of iterations is 30, learning rate $\alpha =10^{-4}$, momentum parameters $\beta_{1}=0.9$, $\beta_{2}=0.999$, weighting decay factor $\lambda =10^{-5}$, batch size = 32. The Adam optimizer was also applied in segmentation. The segmentation models were trained with the following parameters: the number of iterations is 50, learning rate $\alpha =10^{-4}$, weighting decay factor $\lambda =10^{-2}$, batch size = 2.

The classification performance was evaluated by the sensitivity (SEN), specificity (SPE), precision (PRE), F1-score, and accuracy (ACC) metrics defined below:

(3) $$\begin{array}{cc} \mathrm{SEN}= & \;\frac{\mathrm{TP}}{\mathrm{TP}+\mathrm{FN}} \end{array}$$

(4) $$\begin{array}{cc} \mathrm{SPE}= & \;\frac{\mathrm{TN}}{\mathrm{TN}+\mathrm{FP}} \end{array}$$

(5) $$\begin{array}{cc} \mathrm{PRE}= & \;\frac{\mathrm{TP}}{\mathrm{TP}+\mathrm{FP}} \end{array}$$

(6) $$\begin{array}{cc} F1= & \;\frac{2\cdot \mathrm{PRE}\cdot \mathrm{SEN}}{\mathrm{PRE}+\mathrm{SEN}} \end{array}$$

(7) $$\begin{array}{cc} \mathrm{ACC}= & \;\frac{\mathrm{TP}+\mathrm{TN}}{\mathrm{TP}+\mathrm{FP}+\mathrm{TN}+\mathrm{FN}} \end{array}$$

in which TP, TN, FP, FN are short for the number of true positive, true negative, false positive, and false negative cases, respectively. The numbers are counted based on the multi-classification confusion matrix.

The segmentation performance was evaluated by DSC, Hausdorff distance (HD) and average symmetric surface distance (ASSD), defined as follows:

(8) $$\begin{array}{cc} \mathrm{DSC}= & \;\frac{2\left|G\cap M\right|}{\left|G|+|M\right|} \end{array}$$

(9) $$\begin{array}{cc} \mathrm{HD}= & \;\max\left[\max_{p\in \partial R_{G}}d\left(p,\partial R_{M}\right)+\max_{p\in \partial R_{M}}d\left(p,\partial R_{G}\right)\right] \end{array}$$

(10) $$\begin{array}{cc} \mathrm{ASSD}= & \;\frac{1}{2}\left[\frac{1}{\left|\partial R_{G}\right|}\sum_{p\in \partial R_{G}}d\left(p,\partial R_{M}\right)+\frac{1}{\left|\partial R_{M}\right|}\sum_{p\in \partial R_{M}}d\left(p,\partial R_{G}\right)\right] \end{array}$$

where *G* denotes the binary segmentation ground truth, and *M* denotes the binary predicted segmentation mask. The operation | · | represents the enclosed area of the related region. $\partial R_{G}$ and $\partial R_{M}$ denote the region boundary of *G* and *M*, respectively. $d(p,\partial R_{G})$ and $d(p,\partial R_{M})$ denote the shortest Euclidean distance from a point *p* to $\partial R_{G}$ and $\partial R_{M}$, respectively.

## 3. Results

### 3.1. Classification

We first trained multiple Masked-ResNet-DS models with different numbers of layers and selected the optimal classification backbone. The highest classification accuracy on the validation set across 50 epochs is shown in Table 2. As ResNet18 achieves the highest validation accuracy, it was selected as the backbone of the classification model in the remaining experiments.

Two major innovations are introduced in the classification workflow: (1) The Dice loss supervising the CAM [Equation (2)] and (2) masking of the last feature map in the classification task. Specifically, three classification models were implemented to illustrate the benefits of these two innovations:

- NoMask-ResNet: a baseline ResNet without using any masks in average pooling.
- Masked-ResNet-NDS: ResNet with ground truth masks used in average pooling in the training stage. In the testing stage, the data passes through the network twice, as described in Section 2.2. In this model, the CAM was not supervised by the Dice loss in the training stage.
- Masked-ResNet-DS: same as Masked-ResNet-NDS, except that the CAM was supervised by the Dice loss in the training stage.

The comparison between Masked-ResNet-NDS and Masked-ResNet-DS quantifies the contribution of Innovation (1), whereas the comparison between NoMask-ResNet with the remaining two models quantifies the contribution of Innovation (2).

Table 3 compares the classification performance of NoMask-ResNet, Masked-ResNet-NDS and Masked-ResNet-DS and also with three existing single-task classification methods: VGG16, SPP-VGG and MSP-VGG [18]. Additionally, we also compared with two multi-task segmentation-classification model: the MB-DCNN by Xie et al. [23] and RCCM-Net by Gan et al. [28]. We note that the sensitivity, specificity, precision and F1-score for VGG16, SPP-VGG and MSP-VGG are from Ma et al. [18], in which they performed 5-fold cross-validation and reported five sets of results. The range of each metric was listed in Table 3. The classification accuracy of VGG16 and SPP-VGG was not reported by Ma et al. [18], whereas the accuracy of MSP-VGG was reported for the entire dataset. Additionally, we note that RCCM-Net [28] was not reproduced in this study. The classification metrics reported in Table 3 were taken directly from Gan et al. [28], whose experiments involved a dataset with 1270 images that substantially overlaps with ours. However, since the test images differed, the RCCM-Net results are not directly comparable to those of the other methods in Table 3. Nevertheless, the inclusion of RCCM-Net remains informative, as it was evaluated on a closely related dataset.

Both Masked-ResNet-NDS and Masked-ResNet-DS outperformed NoMask-ResNet in all performance metrics, indicating that focusing on plaque features by average pooling using the ground truth mask benefits classification performance. Additionally, Masked-ResNet-DS outperformed Masked-ResNet-NDS in all performance metrics, showing that CAM supervision by the Dice loss function improves classification performance. Specifically, we note that Masked-ResNet-DS achieved 100% specificity and precision for EG plaque classification. The mean sensitivity, specificity, precision, F1-score and accuracy of both Masked-ResNet-NDS and Masked-ResNet-DS were higher than existing methods.

**Figure 2:**
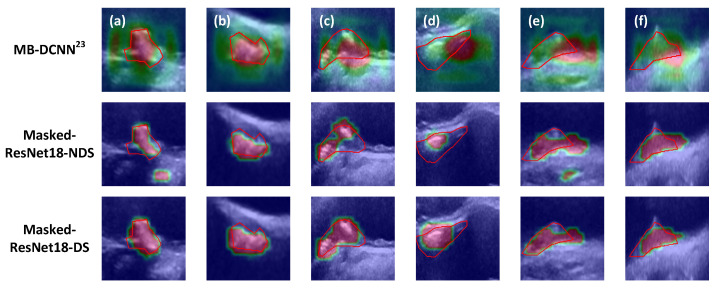
CAMs generated for six example plaque images in the test set, color-coded and superimposed on the 2DUS images (warmer colors indicate stronger CAM activation, while cooler colors indicate weaker activation). Each column corresponds to the same plaque example with identical manual segmentation ground truth, represented by the red contours. Columns (**a**,**b**): echogenic (EG) plaques; columns (**c**,**d**): intermediate (IM) plaques; columns (**e**,**f**): echolucent (EL) plaques. The three rows, from top to bottom, show the CAMs generated by MB-DCNN [23], Masked-ResNet18-NDS, and Masked-ResNet18-DS, respectively.

Figure 2 compares the CAMs generated for six example test set plaques by Masked-ResNet-NDS and Masked-ResNet-DS to illustrate the effect of Dice loss supervision $L_{\mathrm{Dice}}$ [Equation (2)] on the quality of the CAMs. The CAMs from the first iteration of Masked-ResNet-NDS and Masked-ResNet-DS were shown, as they guide averaging pooling in the second iteration, directly influencing classification performance. In Figure 2, the CAMs are superimposed on the 2DUS images with the manually segmented boundaries represented in red. Each column shows the same plaque example, with the six examples labeled from (a) to (f). (a) and (b) are EG plaques; (c) and (d) are IM plaques; (e) and (f) are EL plaques. Comparison between the CAMs produced by Masked-ResNet-NDS and Masked-ResNet-DS showed that the CAMs generated by Masked-ResNet-DS had a larger overlap with the manually segmented boundaries, thereby demonstrating the effectiveness of Dice loss supervision in generating the CAMs. We further compared the CAMs generated by our approaches with those by MB-DCNN [23], another network that uses CAM to guide segmentation. Figure 2 shows that MB-DCNN was less able to focus on the plaques.

### 3.2. Segmentation

An important innovation is the use of CAMs as the mask prompt for MedSAM. The performance improvement attributable to this innovation was quantified by comparing the baseline MedSAM (Base-MedSAM) and CAM-MedSAM. While both models were trained by the ground truth segmentation with the plaque ultrasound image as an input, CAM-MedSAM was additionally provided with the CAM mask prompt generated by Masked-ResNet-DS.

**Figure 3:**
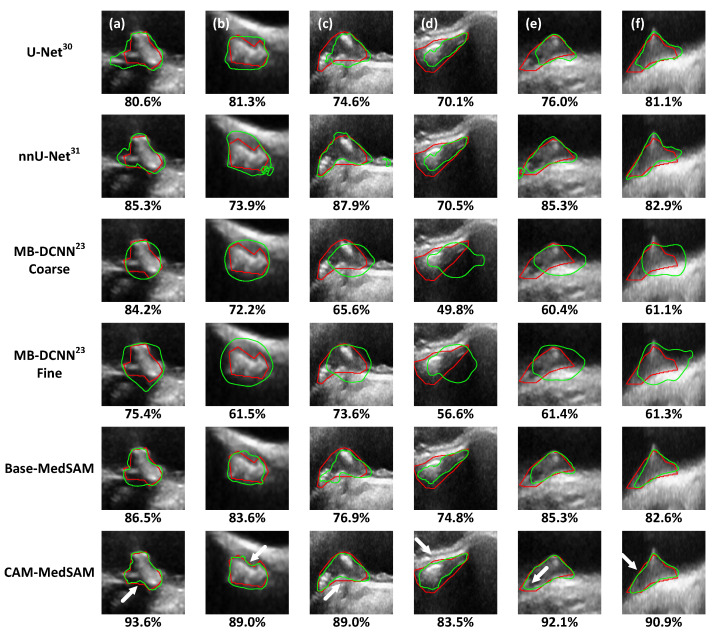
Segmented boundaries by different models on the testing set. (i) in Case (**a**,**b**), the subjects are from the echogenic (EG) category. (ii) in Case (**c**,**d**), the subjects are from the intermediate (IM) category. (iii) in Case (**e**,**f**), the subjects are from the echolucent (EL) category. The red and green contours represent the boundaries from the manual segmentation and the network prediction, respectively. White arrows point to irregular boundaries, where CAM-MedSAM yields more accurate delineation than Base-MedSAM.

Table 4 reports the DSC, HD and ASSD of segmented plaque boundaries produced by Base-MedSAM, CAM-MedSAM, two existing single-task segmentation models: U-Net [29], nnU-Net [30], and two multi-task segmentation-classification pipeline MB-DCNN [23] and RCCM-Net [28]. For MB-DCNN, we report the results for both the initial coarse segmentation and the fine segmentation, the latter generated by the guidance of the CAM from the classification network. As we did not reproduce RCCM-Net, only results from the paper are tabulated. Table 4 list only the overall mean values for the subset consisting of 1270 images, as mean values for individual plaque categories were not reported by Gan et al. [28] Figure 3 shows the segmentation results for the same example subjects shown in Figure 2, with manual and predicted segmentations represented in red and green, respectively. The DSC value for the boundary on each image is listed below the corresponding figures.

U-Net achieved an average DSC of 80.7% in the test set, as shown in Table 4, localizing plaques successfully but struggling with unclear boundaries, as shown in Figure 3. The average DSC was improved to 83.0% by using nnU-Net. The improvement can also be visualized in Figure 3, in particular in Figure 3c. However, nnU-Net did not segment irregular boundaries well, such as Figure 3a,b. Base-MedSAM improves the DSC to 84.4%, leveraging MedSAM’s robust segmentation capabilities to better capture plaque shapes (e.g., Figure 3a, DSC = 86.5%). CAM-MedSAM was provided with CAM prompts and provided a further 2.2% increase in average DSC of the test set compared to Base-MedSAM. Additionally, the mean DSC achieved by CAM-MedSAM is statistically higher than the four existing models we reproduced and compared ($2.77\times 10^{-83}\leq p\leq 2.58\times 10^{-19}$). Note that our algorithm cannot be directly compared with RCCM-Net as the two studies involve different images; therefore, the corresponding statistical test was not performed. The statistically significant improvement of CAM-MedSAM over Base-MedSAM ($p=2.95\times 10^{-10}$) demonstrates the benefits of using CAM as the mask prompt on segmentation performance. Compared to Base-MedSAM, CAM-MedSAM improves segmentation accuracy at regions pointed to by arrows in Figure 3, many of which are curved boundaries with higher complexity in their shapes.

### 3.3. Performance in Challenging Cases

**Figure 4:**
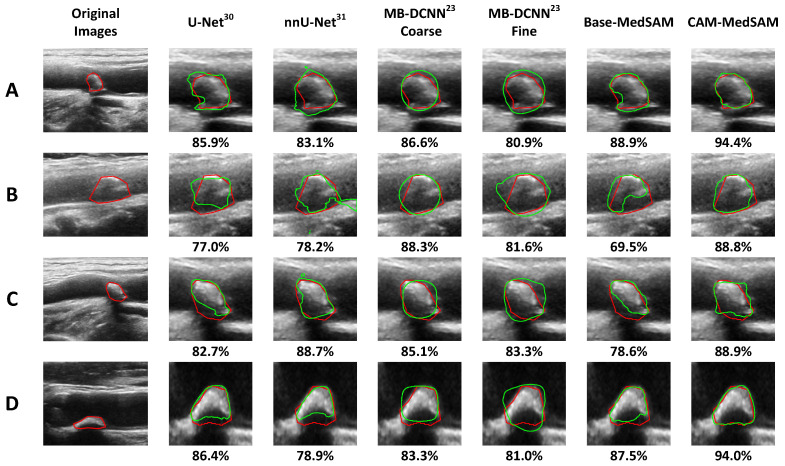
Segmentation results from different models for four challenging cases with high-grade stenosis and/or marked calcification, labeled (**A**–**D**). Each row shows the original image and the segmentation results of competitive methods for an example. Red and green contours represent the manual and algorithm segmentations, respectively. The percentages below the images indicate the Dice coefficients (DSC) between the manual and algorithm segmentations.

In high-grade stenosis, the narrowed lumen reduces flexibility in probe positioning and optimal insonation angle, making boundary visibility more sensitive to small acquisition changes. In addition, severe stenosis may co-occur with calcification, which can attenuate or reflect the ultrasound beam, producing acoustic shadowing that obscures distal structures and further degrades boundary visualization. We therefore selected four representative examples with high-grade stenosis and/or marked calcification from the test set to illustrate that the proposed framework performs robustly under these challenging imaging conditions. These examples are shown in Figure 4, with classification results summarized in Table 5. All four plaques were labeled as echogenic (EG) by the expert clinician. Masked-ResNet18-NDS and Masked-ResNet18-DS correctly classified all cases, whereas MB-DCNN and NoMask-ResNet18 were less accurate. For segmentation, Figure 4 shows the proposed CAM-MedSAM achieved consistently higher DSC compared than competing models. Collectively, these results support that the Dice-supervised CAM improves both the classification and segmentation accuracy in clinically challenging subsets, reinforcing the applicability of our cascaded framework to real-world carotid ultrasound scenarios involving severe stenosis and/or calcification.

## 4. Discussion

Both plaque classification and segmentation from carotid ultrasound images fundamentally rely on the models’ ability to accurately focus on the plaque. Segmentation explicitly delineates the spatial extent of the plaque, whereas classification requires extracting discriminative echogenic features from the plaque region. Therefore, allowing information flow between the classification and segmentation tasks would benefit the performance of both tasks.

In particular, instead of using the ground truth plaque boundaries solely for training the segmentation model, we propose leveraging these boundaries to guide the classification model. Specifically, average pooling is performed only within the regions masked by the ground truth boundaries in the training stage, allowing the classifier to focus on the plaque regions during feature aggregation. Since the ground truth boundaries are not available during inference, we propose a two-iteration classification inference approach that generates the CAM in the first iteration of the classifier and applies the resulting CAM for average pooling in the second iteration. Table 3 demonstrates the success of this approach by showing that the masked ResNets with and without Dice loss supervision (i.e., Masked-ResNet-NDS and Masked-ResNet-DS) perform substantially better than the base model that does not involve the ground truth mask in average pooling (i.e., NoMask-ResNet). The classification performance of the Masked-ResNet-DS model is better than that of the Masked-ResNet-NDS model, thereby demonstrating that CAM supervision by Dice loss improves the classification accuracy.

We compared the performance of the proposed Masked-ResNet-DS model with methods presented by Ma et al. [18], which employed 5-fold cross-validation on the same dataset. Due to the unavailability of information on the specific subjects included in each fold involved in their study, direct comparison cannot be performed. Instead, we split the data into training, validation and testing sets in the same partition ratio as [18] and compare our results with all five sets of results reported for the 5-fold cross-validation studies by [18]. In Table 3, the performance metrics of the VGG16, SPP-VGG and MSP-VGG models across five folds are reported as ranges. Our model outperforms these three existing frameworks in their best performing fold, except in sensitivity for the EG plaques. This superiority further validates the effectiveness of the dual mechanism of average pooling masking and CAM supervision in classification.

The use of the supervised CAM as mask prompts in the training and inference of the MedSAM significantly enhances segmentation performance compared to existing methods and Base-MedSAM, as demonstrated in Table 4. Figure 3 demonstrates the improved segmentation precision of CAM-MedSAM. This illustration shows that CAM-MedSAM was more able than Base-MedSAM in segmenting challenging regions, including concave sections of the plaque boundaries.

In comparison with the segmentation-classification pipeline MB-DCNN [23], our model demonstrates superior performance in both classification and segmentation of carotid artery plaques. The MB-DCNN model employs a cascaded approach comprising a coarse segmentation network, a classification network, and a fine segmentation network, all based on complex architectures. Specifically, the coarse segmentation network, built on DeepLab V3+, fails to converge well on our dataset after 500 epochs of training, resulting in average DSCs of 72.9%, 70.3%, and 72.6% on the training, validation and testing sets, respectively. As a result, the coarse segmentation network generates suboptimal segmentation masks compared to simpler models, such as U-Net (as shown in Figure 3 and Table 4). For classification, MB-DCNN relies on the Xception network, which, due to its large parameter count, overfits the training set. While achieving perfect training accuracy (100% for each category), the Xception network had low validation and testing accuracies (average classification accuracies of 52.1% and 43.5%, respectively). Additionally, the inaccurate coarse segmentation masks provide limited guidance for classification, and the absence of CAM supervision during the classification phase results in CAMs that fail to localize the plaque region accurately, further lowering classification performance. For fine segmentation, MB-DCNN again employs DeepLab V3+. As the CAM could not localize the plaque accurately (Figure 2), the fusion of the CAM with the encoded features in the fine segmentation network even decreases the segmentation performance compared to the coarse segmentation results, leading to average DSCs of 71.9%, 69.5%, and 70.9% on the training, validation and testing sets, respectively. In contrast, our CAM can localize plaques accurately with the CAM supervised by the ground truth via Dice loss (Figure 2). Using the supervised CAM as a prompt to the MedSAM, we were able to improve MedSAM’s segmentation performance.

The multi-task RCCM-Net [28] was trained and tested on a subset of images in the current dataset. Their approach leverages a shared encoder and incorporates two modules to enhance classification and segmentation performance: the Region Confidence Module (RCM) and the Category Confidence Module (CCM). RCM enhances plaque focus to improve classification performance, whereas CCM weights the segmentation loss function based on classification certainty. Although the reported results for RCCM-Net and our proposed model are not directly comparable due to differences in the datasets used in the respective studies, the higher accuracy achieved by our approach (Table 3) indicates substantially better classification performance. A plausible explanation for this improvement is the use of CAM supervision via Dice loss in our method. While RCM highlights plaque-prone regions based on feature maps from the segmentation model, its feature maps are not directly supervised. Therefore, there is no guarantee that plaques are consistently and accurately highlighted for subsequent classification. Secondly, while it is useful to rate the reliability of ground truth boundaries and use this rating to train segmentation models, the use of classification certainty generated by CCM to rate segmentation reliability is questionable. Improved assessment of manual segmentation reliability can be based on boundary standard deviation from repeated segmentations, as proposed in several studies [31,32]. Before such an assessment metric has been developed and thoroughly validated, manual segmentation for different plaques should be considered equally reliable during training, just as in CAM-MedSAM proposed in this study.

The proposed cascaded framework can be integrated into clinical workflows as an automated tool for plaque echogenicity classification and for TPA measurements. Risk stratification has already been done based on plaque echogenicity [33] and TPA quantification [4]. TPA progression enables a “treat-the-artery” paradigm, where therapy is guided by plaque change rather than traditional risk-factor targets [34]. Serial TPA identifies plaque progressors for targeted treatment intensification to halt progression or induce regression while avoiding unnecessary escalation in stable or regressing patients. Additionally, TPA measurements have been used to evaluate vascular risk factors and the effect of treatments on plaque progression/regression [35]. The automated nature of the proposed framework will reduce the time required and the variability associated with manual plaque classification and delineation.

Although we demonstrated that the proposed framework outperformed existing approaches, this study has several limitations. First, the proposed framework was trained and tested on a dataset acquired at a single institute. Although the dataset is large and encompasses plaques exhibiting diverse echogenic properties, multi-centered investigation is needed to demonstrate the generalizability of the framework to different ultrasound devices and patients with varying degrees of atherosclerosis. Such analysis may require adaptation techniques, such as that used for carotid vessel wall segmentation [36], to improve the generalization ability of the framework to image data acquired in different centers.

Second, the classification labels of the dataset were provided by a single observer. Although the observer was experienced and demonstrated good intraobserver reproducibility (κ=0.747) [18], further increase in observer reproducibility by consensus labeling from expert observers from multiple institutes would benefit the classification training by further reducing subjective bias in manual annotation.

Third, our segmentation network operates automatically without providing an opportunity for the user to improve the segmentation. The segmentation performance could benefit by allowing users to input interactive prompts to improve MedSAM segmentation iteratively. However, it would be time-consuming for an observer to check the automatically segmented boundaries for thousands of images. This challenge can be addressed by a “rejection” network, such as the one proposed by Kou et al. [37], which rejects or flags potentially inaccurate boundaries for observer intervention. However, the rejection threshold for plaque segmentation would require dedicated tuning to balance plaque segmentation accuracy and time required for intervention.

Fourth, several technical challenges remain for integrating the proposed framework into routine clinical workflows. Although the per-image inference time is short in the current study (classification: 0.1 s, segmentation: 7.5 s), it may increase in clinics with limited computing resources. Cloud-based or edge-computing deployment could reduce local hardware requirements and enable near real-time inference on standard clinical workstations. Additionally, the results were generated in a deep learning environment with command-line interface; a more user-friendly interface will be needed to support clinical uses.

## 5. Conclusions

This study presents a cascaded framework for carotid plaque classification and segmentation from 2D ultrasound images, integrating a ResNet-based classifier with the MedSAM. By enabling information flow between the two tasks, the framework ensures that both classification and segmentation more effectively focus on plaque regions—critical for identifying plaque echogenicity and delineating boundaries. The key to this integration is the use of CAMs, first as masks to help the classifier focus on the plaque during inference, and second as mask prompts for MedSAM. The CAMs are supervised using a Dice loss to ensure they cover the whole plaque region. In addition, classification training is guided by average pooling restricted to ground truth plaque areas, while a two-iteration inference strategy allows CAM-guided focus without requiring annotations at test time. Experimental results demonstrated that this approach substantially improved both classification accuracy and segmentation quality over baseline methods. Overall, the proposed framework offers a simple and effective dual-task pipeline that advances the integration of image classification and CAM-prompt-based segmentation in clinical ultrasound imaging. Future work will be required to validate the framework in multi-center clinical studies and develop a user-friendly interface to facilitate translation from research to clinical practice.

## Figures and Tables

**Table 1:** Data partition details of the selected dataset for training, validation and testing.

Types of Plaques	Images	Training Set	Validation Set	Testing Set
Echogenic (EG)	335	234	32	69
Intermediate (IM)	405	275	40	90
Echolucent (EL)	723	516	72	135
Total	1463	1025	144	294

**Table 2:** Highest classification accuracy on validation set for four ResNet models.

Model	Classification Accuracy (%)
ResNet18	87.0
ResNet34	86.8
ResNet50	85.4
ResNet101	85.2

**Table 3:** Classification results comparison among MSP-VGG [18], NoMask-ResNet18, Masked-ResNet18-NDS and Masked-ResNet18-DS models on the testing set. SEN: sensitivity, SPE: specificity, PRE: precision, F1: F1-scores, ACC: Accuracy. The hyphen (“-”) indicates that the metric was not reported in the corresponding publication. The top-performing metrics are highlighted in bold.

Metrics	Model	EG (%)	IM (%)	EL (%)	Mean (%)
SEN	VGG16 [18]	70.8–84.6	63.4–73.3	89.0–96.0	75.2–82.8
	SPP-VGG [18]	91.7–98.5	76.5–91.1	87.4–96.0	86.1–93.0
	MSP-VGG [18]	92.5–**98.6**	81.8–91.1	89.6–96.0	90.9–94.1
	MB-DCNN [23]	18.8	28.9	65.9	37.9
	NoMask-ResNet18	72.5	67.8	94.1	78.1
	Masked-ResNet18-NDS	92.8	93.3	98.5	94.9
	Masked-ResNet18-DS	94.2	**96.7**	**98.5**	**96.5**
SPE	VGG16 [18]	96.5–99.5	84.8–92.9	77.3–83.1	86.9–90.8
	SPP-VGG [18]	97.6–97.9	92.5–97.2	86.5–94.9	92.4–96.1
	MSP-VGG [18]	98.1–99.5	93.8–96.3	90.7–95.7	95.0–96.6
	MB-DCNN [23]	79.6	79.4	50.9	69.9
	NoMask-ResNet18	98.2	90.7	79.2	89.4
	Masked-ResNet18-NDS	99.1	97.1	96.9	97.7
	Masked-ResNet18-DS	**100.0**	**97.2**	**98.1**	**98.4**
PRE	VGG16 [18]	89.1–98.0	61.9–82.5	80.6–86.0	80.2–85.4
	SPP-VGG [18]	92.5–94.3	80.8–89.3	86.4–94.7	87.1–92.3
	MSP-VGG [18]	94.1–98.6	84.9–88.7	90.4–95.9	91.3–92.9
	MB-DCNN [23]	22.0	38.2	53.3	37.9
	NoMask-ResNet18	92.6	76.3	79.4	82.8
	Masked-ResNet18-NDS	97.0	93.3	96.4	95.6
	Masked-ResNet18-DS	**100.0**	**93.5**	**97.8**	**97.1**
F1	VGG16 [18]	81.6–88.7	62.7–77.7	84.6–89.2	77.6–83.9
	SPP-VGG [18]	93.0–95.5	78.5–88.2	88.2–94.0	86.5–92.6
	MSP-VGG [18]	93.9–96.5	85.1–89.7	91.6–94.3	91.3–93.5
	MB-DCNN [23]	20.3	32.9	58.9	37.4
	RCCM-Net [28]	-	-	-	85.4
	NoMask-ResNet18	81.3	71.8	86.1	79.7
	Masked-ResNet18-NDS	94.9	93.3	97.4	95.2
	Masked-ResNet18-DS	**97.0**	**95.1**	**98.1**	**96.7**
ACC	MSP-VGG [18]	**95.9**	87.6	92.7	92.1
	MB-DCNN [23]	18.8	28.9	65.9	37.9
	RCCM-Net [28]	91.1	92.3	71.7	85.0
	NoMask-ResNet18	72.5	67.8	94.1	78.1
	Masked-ResNet18-NDS	92.8	93.3	98.5	94.9
	Masked-ResNet18-DS	94.2	**96.7**	**98.5**	**96.5**

**Table 4:** Segmentation performance of our method compared to existing methods. The mean and standard deviation of the DSC (expressed in percentage), HD and ASSD (expressed in mm) are reported for each plaque category [i.e., echogenic (EG), intermediate (IM) and echolucent (EL)] and for the overall testing set. The hyphen (“-”) indicates that the metric was not reported in the corresponding publication. The top-performing metrics are highlighted in bold.

Metrics	Model	EG	IM	EL	Overall
DSC	UNet [29]	81.8 ± 7.3	80.1 ± 9.8	80.6 ± 8.5	80.7 ± 8.7
	nnUNet [30]	81.9 ± 7.7	83.6 ± 7.4	83.1 ± 6.0	83.0 ± 6.9
	MB-DCNN (Coarse) [23]	79.8 ± 9.0	71.7 ± 10.7	68.1 ± 10.2	72.2 ± 10.1
	MB-DCNN (Fine) [23]	73.1 ± 9.8	73.5 ± 9.6	68.1 ± 7.5	70.9 ± 9.1
	RCCM-Net [28]	-	-	-	84.9 ± 0.4
	Base-MedSAM	85.2 ± 6.2	82.9 ± 9.5	85.1 ± 6.2	84.4 ± 7.4
	CAM-MedSAM	**87.5 ± 4.2**	**85.4 ± 6.9**	**87.1 ± 5.4**	**86.6 ± 5.7**
HD	UNet [29]	0.78 ± 0.56	1.92 ± 1.51	1.59 ± 1.36	1.50 ± 1.34
	nnUNet [30]	0.81 ± 0.80	1.63 ± 1.12	1.48 ± 1.20	1.37 ± 1.13
	MB-DCNN (Coarse) [23]	0.82 ± 0.51	2.80 ± 1.88	2.24 ± 1.73	2.08 ± 1.75
	MB-DCNN (Fine) [23]	1.16 ± 0.55	2.41 ± 1.65	2.28 ± 1.37	2.06 ± 1.42
	RCCM-Net [28]	-	-	-	0.75 ± 0.05
	Base-MedSAM	0.66 ± 0.56	1.42 ± 1.36	1.21 ± 1.02	1.15 ± 1.10
	CAM-MedSAM	**0.46 ± 0.19**	**0.86 ± 0.49**	**0.79 ± 0.52**	**0.73 ± 0.48**
ASSD	UNet [29]	0.22 ± 0.12	0.32 ± 0.21	0.25 ± 0.18	0.27 ± 0.18
	nnUNet [30]	0.21 ± 0.18	0.25 ± 0.15	0.20 ± 0.10	0.22 ± 0.14
	MB-DCNN (Coarse) [23]	0.26 ± 0.16	0.55 ± 0.33	0.47 ± 0.27	0.45 ± 0.29
	MB-DCNN (Fine) [23]	0.39 ± 0.15	0.53 ± 0.33	0.50 ± 0.19	0.49 ± 0.24
	RCCM-Net [28]	-	-	-	0.27 ± 0.004
	Base-MedSAM	0.17 ± 0.09	0.24 ± 0.19	0.17 ± 0.10	0.19 ± 0.14
	CAM-MedSAM	**0.13 ± 0.05**	**0.16 ± 0.06**	**0.13 ± 0.05**	**0.14 ± 0.06**

**Table 5:** Classification results for four challenging test cases with high-grade stenosis and/or marked calcification from MB-DCNN [23], NoMask-ResNet18, Masked-ResNet18-NDS, and Masked-ResNet18-DS. These cases are referred to as Plaques A, B, C and D according to Figure 4. All plaques are labeled echogenic (EG) by the expert clinician.

Models	Plaque			
	A	B	C	D
MB-DCNN [23]	IM	EL	EL	EL
NoMask-ResNet18	EL	EG	IM	EL
Masked-ResNet18-NDS	EG	EG	EG	EG
Masked-ResNet18-DS	EG	EG	EG	EG

## Data Availability

The data supporting the findings of this study are available within the article. Further inquiries may be directed to the corresponding author.
